# Novel Biomarker for Antimitochondrial Antibodies in Systemic Sclerosis Patients

**DOI:** 10.1155/jimr/2726162

**Published:** 2026-07-05

**Authors:** Elvira Favoino, Silvia De Santis, Vasiliki Liakouli, Giovanna Barbuti, Sabina Piccolo, Ada Corrado, Marta Vomero, Luca Navarini, Marcella Prete, Patrizia Leone, Vito Racanelli, Rosa Daniela Grembiale, Piero Ruscitti, Francesco Paolo Cantatore, Francesco Ciccia, Roberto Giacomelli, Federico Perosa

**Affiliations:** ^1^ Laboratory of Cellular and Molecular Immunology, Department of Interdisciplinary Medicine, University of Bari Medical School, Bari, Italy, uniba.it; ^2^ Rheumatology Section, Department of Precision Medicine, University of Campania “Luigi Vanvitelli”, Naples, Italy, unina2.it; ^3^ Department of Biomedical Sciences and Human Oncology, University of Bari Medical School, Bari, Italy, uniba.it; ^4^ Rheumatology Unit, Department of Medical and Surgery Sciences, University of Foggia, Foggia, Italy, unifg.it; ^5^ Clinical and Research Section of Rheumatology and Clinical Immunology, Fondazione Policlinico Campus Bio-Medico, Via Álvaro del Portillo 200, Rome, 00128, Italy, unicampus.it; ^6^ Rheumatology and Clinical Immunology, Department of Medicine, School of Medicine, University of Rome “Campus Biomedico”, Rome, Italy, unicampus.it; ^7^ Internal Medicine Unit, Department of Interdisciplinary Medicine, University of Bari Medical School, Bari, Italy, uniba.it; ^8^ Centre for Medical Sciences, Santa Chiara Hospital, Provincial Health Care Agency (APSS), University of Trento and Internal Medicine Division, Trento, Italy, apss.tn.it; ^9^ Rheumatology Research Unit, Department of Health Sciences, University of Catanzaro Magna Graecia, Catanzaro, Italy, unicz.it; ^10^ Rheumatology Unit, Department of Biotechnological and Applied Clinical Sciences, University of L’Aquila, L’Aquila, Italy, univaq.it; ^11^ Rheumatic and Systemic Autoimmune Diseases Unit, Department of Interdisciplinary Medicine, University of Bari Medical School, Bari, Italy, uniba.it

**Keywords:** anti-CENP-A antibodies, antimitochondrial antibodies, biomarker risk, primary biliary cholangitis, systemic sclerosis

## Abstract

**Objective:**

We previously identified, using a synthetic peptide, namely peptide 4.33 (p4.33), a subgroup of anti‐CENP‐A antibodies (Abs) recognizing an epitope shared between the CENP‐A region spanning amino acids 1‐17 (Ap1‐17) and the E2 component of the mitochondrial pyruvate dehydrogenase complex (PDC‐E2), the major mitochondrial target autoantigen in primary biliary cholangitis (PBC). Here, we evaluated whether anti‐p4.33 Ab positivity may be associates with a higher prevalence of antimitochondrial Ab (AMA) in systemic sclerosis (SSc) patients.

**Methods:**

Serum samples from 145 anti‐CENP^pos^ SSc patients were tested for anti‐CENP‐A, ‐Ap1‐17, and ‐p4.33 Abs by ELISA. Subsequently, 32 anti‐Ap1‐17^pos^/p4.33^pos^ and 32 anti‐Ap1‐17^pos^/p4.33^neg^ were randomly selected and tested for AMA by immunoblotting.

**Results:**

Of 145 anti‐CENP^pos^ patients, 128 (88.2%) were anti‐CENP‐A^pos^ patients, of which 103 (80.5%) were positive for anti‐Ap1‐17 Abs and 66 (51.6%) were positive for anti‐p4.33 Abs. Of 32 selected anti‐Ap1‐17^pos^/p4.33^pos^ patients, 15 (46.9%) were also positive for AMA targeting PDC‐E2 and/or the branched chain 2‐oxo acid dehydrogenase complex (BCOADC‐E2). In the Ap1‐17^pos^/p4.33^neg^ group, AMA were detected in only two patients (6.25%). AMA positivity was statistically different between groups. Anti‐p4.33 Ab levels were directly associated more than anti‐Ap1‐17 Ab levels with AMA levels. Sequence homology analyses demonstrated that anti‐p4.33 Abs recognize an epitope (kPsaP) strongly overlapping with those of Ap1‐17, PDC‐E2, and BCOADC‐E2, also expressed by viral and bacterial proteins.

**Conclusions:**

Anti‐p4.33 Abs may be a useful biomarker to define a subset of SSc patients with a higher prevalence of AMA. Our findings also support the hypothesis that pathogens can trigger autoimmunity.

## 1. Introduction

Systemic sclerosis (SSc) is a heterogeneous autoimmune connective tissue disease characterized by vascular abnormalities, immune dysfunction, and fibrosis [1]. Although the etiology of SSc is still unknown, factors that may contribute to the development of the disease include genetic background, environmental factors, and molecular mimicry.

SSc is characterized by the presence of antinuclear antibodies (ANA), detected in up to 95% of patients. ANAs are directed against a broad spectrum of self‐antigens, including DNA topoisomerase‐I (topo‐I) and centromeric‐associated proteins (CENP). ANA subtypes have been linked to different clinical features [2, 3]. For instance, anti‐CENP antibodies (Abs) are associated with limited skin involvement (lcSSc) and pulmonary arterial hypertension [4, 5], while anti‐topo‐I Abs are associated with diffuse skin involvement (dcSSc) and pulmonary fibrosis [6]; anti‐U3‐RNP Abs with dcSSc and skeletal muscle involvement [7]; and anti‐RNA polymerase III Abs with dcSSc and scleroderma renal crisis [8].

SSc may overlap with other autoimmune diseases, including systemic lupus erythematosus, rheumatoid arthritis, Sjögren syndrome, and organ‐specific diseases, such as Hashimoto’s thyroiditis [9] and primary biliary cholangitis (PBC).

PBC is a chronic, slowly progressing cholestatic liver disease that, without proper treatment, can lead to cirrhosis and liver failure. PBC is often asymptomatic during the early stages, making a prompt diagnosis challenging. PBC is the most common liver disease in SSc, with a prevalence estimated at about 2%–3% [10, 11] but higher in lcSSc. PBC is characterized by the presence of autoantibodies targeting mitochondrial antigens (antimitochondrial Abs [AMAs]) in 90%–95% of patients, and AMA positivity is predictive of PBC development in asymptomatic patients [6].

AMA is detected in 2.5%–25% of SSc patients and in up to 30% of anti‐CENP^pos^ SSc patients [12–14].

In a previous work, we demonstrated for the first time that some SSc patients‐derived anti‐CENP Ab were directed against the amino‐terminal region of CENP‐A, spanning amino acids 1‐17 (Ap1‐17), one of the two dominant epitopes of anti‐CENP‐A Abs [15]. They cross‐reacted with the major AMA target in PBC, namely, the E2 component of the mitochondrial pyruvate dehydrogenase complex (PDC‐E2), indicating that these Ab and AMA are the same Ab population in a subset of SSc patients [16]. The results were obtained by panning a phage display peptide library with anti‐Ap1‐17 Abs purified from an SSc/PBC patient, which led to the isolation of anti‐Ap1‐17 Ab‐specific phage clones [17].

Here, we evaluated whether the presence of Abs to peptide 4.33 (p4.33), expressed by one of the above‐mentioned Ap1‐17‐specific phage clones, may define a subgroup of patients with a higher prevalence of AMA and greater likelihood of developing PBC.

## 2. Materials and Methods

### 2.1. Serum Samples and Reagents

This retrospective study analyzed serum samples from 145 anti‐CENP^pos^ SSc patients as determined by routine hospital testing using the anti‐CENP‐B ELISA kit (Orgentec Diagnostika GmbH, Germany). Patients were recruited at the Rheumatology Units of the Universities of Bari, Naples, Foggia, L’Aquila, and Rome from 2018–2022 and satisfied the 2013 ACR/EULAR classification criteria. For each patient, data related to gender, age, age at the onset of the first Raynaud phenomenon, disease duration (determined from the onset of Raynaud’s phenomenon), and SSc subset (limited or diffuse according to Mehra et al. [3]) were collected. Serum samples from 57 age‐matched healthy blood donors (HBDs) were used as controls. Ap1‐17/17‐30 peptide, corresponding to the two immunodominant epitopes of CENP‐A and reacting with 96% of CENP‐B Ab‐positive sera [17], and the Rituximab‐specific peptide Rp10‐L [18] were previously isolated and characterized.

### 2.2. Peptides

Peptides were synthesized at Primm (Milan, Italy) with a percentage purity greater than 90%. Individual peptides, as well as the 1:1 mixture of Ap1‐17/Ap17‐30 peptide (Ap1‐17/17‐30), were coupled to keyhole limpet hemocyanin (KLH), as previously described [17]. Rituximab‐specific peptide Rp10‐L was used as a negative control.

### 2.3. Peptide‐Based ELISA

The indirect binding assay to quantify antipeptide Ab levels was performed as previously described [19], with minor modifications. Briefly, plates were incubated with a PBS solution containing 5 μg/mL of KLH‐coupled peptides, o/n at 4°C. After one washing and blockage of free protein‐binding sites, serum samples diluted 1:100 were added to the plate for 3 h at R/T. Antigen‐Ab immunocomplexes were detected by adding HRP‐conjugated anti‐human IgG and OPD. The absorbance was read at 490 nm. Specific binding was determined by subtracting the background binding in wells coated with KLH‐conjugated negative control peptide Rp10‐L from the binding in experimental wells. Serum samples from pt4 were used as a positive control. The levels of Abs in sera were expressed as the percentage of binding compared to the positive control. Samples were tested in duplicate, and experiments were performed three times.

### 2.4. Immunodot

Anti‐M2 Abs were detected using a line‐dot immunoassay from Alphadia (Liver Profile 10 Ag Dot for BlueDiver, Mons, Belgium), as previously described [16]. Every single strip contained 10 dots for the simultaneous detection of Abs against different antigens, including the mitochondrial antigens naïve PDC (nPDC), PDC‐E2, and the E2 component of the branched chain 2‐oxo acid dehydrogenase complex (BCOADC‐E2) and the nuclear antigens sp100 and gp210. Dot intensity was converted into dot arbitrary units (AU) ranging from 0 (negative result) to 100 (highly positive result) using the formula ([intensity of a specific antigen − intensity of the negative control]/[intensity of the positive control − intensity of the negative control]) x 100. Values less than 5 AU were considered negative.

### 2.5. Statistical Analyses

Statistical analyses were performed using SPSS (v.21) and MedCalc (v. 7.6.0.0) software for Windows. Receiver operating characteristic (ROC) analysis was used to define cut‐off values that best discriminate two groups, defined as the point maximizing the Youden index (sensitivity + specificity − 1). Spearman’s rho test was used to analyze the correlation between two continuous variables. The Mann–Whitney *U* test was used for continuous variables when comparing two groups. Chi‐square and Fisher’s exact tests were used to assess the associations between categorical variables. A multivariable analysis was performed to define the relationship between independent variables and the outcome variable. Statistical significance was set at a *p* value < 0.05.

### 2.6. Sequence Analyses

Sequence homology analyses were performed using CLUSTAL OMEGA at EMBL’s European Bioinformatics Institute (https://www.ebi.ac.uk/ Tools/msa/clustalo/). Viral and bacterial proteins containing the antigenic motif were defined using the Swiss‐Prot Protein Sequence Database and the PROSITE tool (https://prosite.expasy.org).

## 3. Results

### 3.1. Relationships Between Anti‐CENP‐A Ab Subsets and AMA

**Figure 1:**
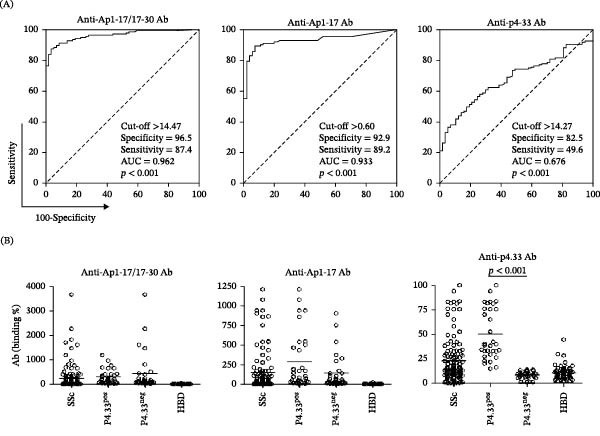
(A) Receiver operating characteristic (ROC) analysis to define the best cut‐off levels of anti‐CENP‐A Ab to Ap1‐17/17‐30, Ap1‐17, and p4.33 to discriminate SSc (*n* = 145) patients from healthy individuals (HBD; *n* = 57). Cut‐off levels are expressed as the percentage of binding compared to the positive control. Area under the curve (AUC), sensitivity, specificity, and exact *p*‐values are indicated. (B) Dot plot representation of ELISA results, where each symbol corresponds to an individual serum sample. Anti‐p4.33^pos^ (*n* = 32) and ‐p4.33^neg^ (*n* = 32) patients randomly selected for AMA testing are shown. The levels of Abs in sera were expressed as the percentage of binding compared to the positive control. ELISA measurements were performed in duplicate and repeated in three independent experiments. Statistical comparisons between anti‐p4.33^pos^ and ‐p4.33^neg^ patients were performed using the Mann–Whitney *U* test; exact *p*‐values are reported for significant results.

In this retrospective study, 145 routinely diagnosed anti‐CENP^pos^ SSc patients were enrolled. To define patients also positive for anti‐CENP‐A Abs, indirect peptide‐based ELISA was performed using the cross‐linked peptides Ap1‐17 and Ap17‐30 (Ap1‐17/17‐30), spanning the CENP‐A‐derived region containing the two immunodominant epitopes. Out of 145 anti‐CENP^pos^ SSc patients, 128 (88.28%) were found to be positive for anti‐CENP‐A Abs. ROC curves were generated to evaluate the ability of anti‐CENP‐A Ab subgroups to discriminate between SSc patients and HBDs. As expected, the accuracy of anti‐Ap1‐17/17‐30 Ab levels in discriminating SSc patients from HBDs (AUC = 0.962, 96.5% specificity, 87.4% sensitivity, *p* < 0.001) was higher than that observed with anti‐Ap1‐17 (AUC = 0.933, 92.9% specificity, 89.2% sensitivity, *p* < 0.001) and anti‐p4.33 (AUC = 0.676, 82.5% specificity, 49.6% sensitivity, *p* < 0.001) Abs (Figure 1A). The optimal cut‐off values to distinguish diseased from healthy patients were 14.47%, 0.60%, and 14.27% for anti‐Ap1‐17/17‐30 Abs, anti‐Ap1‐17 Abs, and anti‐p4.33 Abs, respectively. The optimal anti‐Ap1‐17 and anti‐p4.33 Ab cut‐off points were used to subdivide SSc patients into Ab‐positive and Ab‐negative groups. Of 128 anti‐CENP‐A^pos^ patients, 103 patients (80.5%) were positive for anti‐Ap1‐17 Abs (anti‐Ap1‐17 Ab levels >0.60%) and 66 (51.6%) for anti‐p4.33 Abs (anti‐p4.33 Ab levels >14.27%) (Table 1). Most patients were female (89.70%) and affected by lcSSc (91.41%). Patients’ mean age was 60.83 ± 12.17 years, whereas the mean age at onset of the first symptoms (Raynaud’s phenomenon) was 44.51 ± 16.54 years. The mean disease duration was 21.24 years ± 12.24. As shown in Table 1, there were no differences between anti‐p4.33^pos^ and anti‐p4.33^neg^ patients in sex distribution, age, age at Raynaud’s onset, disease duration, and SSc subset, while anti‐Ap1‐17 Ab levels were comparable between groups (Mann–Whitney *U* test, *p* = 0.080) (Table 1).

**Table 1:** Clinical characteristics of 128 anti‐CENP‐A (1‐17/17‐30) antibodies (Ab)‐positive patients with systemic sclerosis, in the whole cohort, and the anti‐p4.33^pos^ and anti‐p4.33^neg^ subgroups.

Variable	Whole cohort (*n* = 128)	Anti‐Ap1‐17^pos^ (*n* = 103)	
		Anti‐p4.33^pos^ (*n* = 53)	Anti‐p4.33^neg^ (*n* = 50)
Female (*n* [%])	113 (89.70)	49 (92.5)	41 (82)
Age (mean ± SD)	60.83 ± 12.17	62.04 ± 12.35	61.78 ± 12.54
Age at first Raynaud’s phenomenon symptoms (mean ± SD)	44.51 ± 16.54	47.87 ± 15.42	46.62 ± 17.91
Disease duration (mean ± SD)	21.24 ± 12.24	19.59 ± 11.64	20.82 ± 12.96
Limited disease (*n* [%])	117 (91.41)	44 (83)	42 (84)
Anti‐Ap1‐17 Ab (mean ± SD)	142.74 ± 256.24	196.92 ± 301.32	113.85 ± 213.41

Abbreviation: SD, standard deviation.

To explore the association between anti‐p4.33 Abs and AMA, 32 anti‐p4.33^pos^ and 32 anti‐p4.33^neg^ serum samples were randomly selected and tested for AMA. As expected, anti‐p4.33 Ab levels differed significantly between the two groups (Mann–Whitney *U* test, *p*  < 0.001) (Figure 1B). There were no differences between groups in anti‐CENP‐A (Mann–Whitney *U* test, *p* = 0.428) or anti‐Ap1‐17 (Mann–Whitney *U* test, *p* = 0.050) Ab levels (Figure 1B).

**Figure 2:**
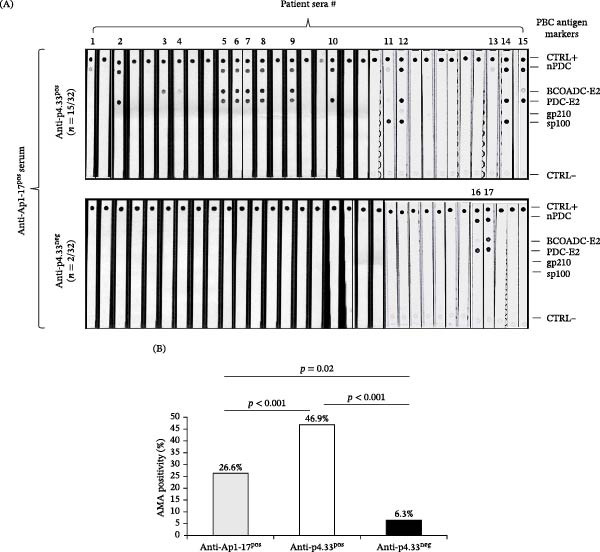
The prevalence of antimitochondrial antibody (AMA) in anti‐p4.33^pos^ patients is higher than in p4.33^neg^ patients. (A) The reactivity of systemic sclerosis anti‐p4.33^pos^ (*n* = 32) and anti‐p4.33^neg^ sera (*n* = 32) with naïve pyruvate dehydrogenase complex (nPDC), PDC, E2 component of the mitochondrial pyruvate dehydrogenase complex (PDC‐E2), and branched chain 2‐oxo acid dehydrogenase complex (BCOADC‐E2) was assessed by the line‐dot immunoassay from Alphadia “Liver Profile 10 Ag Dot.” Each lane corresponds to an individual patient sample. Lanes with antimitochondrial antibody (AMA)‐positive serum were numbered from 1 to 17. (B) Differences in AMA positivity between systemic sclerosis anti‐Ap1‐17^pos^ (*n* = 64), anti‐p4.33^pos^ (*n* = 32), and anti‐p4.33^neg^ (*n* = 32) were analyzed by chi‐square test. Statistical significance was set at *p* < 0.05. Exact *p*‐values are indicated.

AMA were detected in 15 of 32 (46.88%) samples belonging to the first group (Figure 2A) and in only 2 of 32 (6.25%) belonging to the second group (Figure 2A). AMA‐specific Ag targeted by SSc sera were nPDC, detected in 13 patients (lanes #1, 2, 5–15), PDC‐E2, detected in 11 patients (lanes #2, 5–12, 14, 15), and BCOADC‐E2, detected in 8 patients (lanes #3–9, 15) (Figure 2A). In addition to AMA, two patients had Abs against the nuclear antigen sp100 (lanes #11 and 14) and one against sp100 and gp210 (lane #12) (Figure 2A). None of the anti‐p4.33^neg^ patients displayed this type of reactivity (Figure 2A).

The percentage of patients with AMA in the p4.33^pos^ group was markedly higher than in p4.33^neg^ patients (chi‐square test *p* < 0.001) or in the whole cohort of patients positive for anti‐Ap1‐17 Ab (*p* < 0.001) (Figure 2B). In the latter group, AMA prevalence was higher than in p4.33^neg^ patients (*p* = 0.02) (Figure 2B).

To evaluate the relationships between the levels of anti‐p4.33 Abs and those of Abs directed against AMA subgroups, a Spearman’s rho test was performed. As shown in Table 2, anti‐p4.33 Ab levels were significantly correlated with the Ab levels of anti‐nPDC (*R* = 0.403, *p* < 0.001), ‐PDC‐E2 (*R* = 0.408, *p* < 0.001), and ‐BCOADC‐E2 (*R* = 0.391, *p* < 0.001). Although to a lower extent, a similar correlation was also observed between anti‐Ap1‐17 Ab levels and anti‐nPDC (*R* = 0.300, *p* < 0.016), ‐PDC‐E2 (*R* = 0.292, *p* < 0.019), and ‐BCOADC‐E2 (*R* = 0.423, *p* < 0.001) Ab levels. On the other hand, anti‐CENP‐A (Ap1‐17/17‐30) Ab levels barely correlated with anti‐BCOAD Ab levels (*R* = 0.251, *p* = 0.045), while no correlation was observed with Abs to anti‐nPDC and ‐PDC‐E2.

**Table 2:** Anti‐p4.33 antibodies (Ab) levels correlated better than anti‐Ap1‐17 Ab with antimitochondrial Ab (AMA) to the E2 components of the pyruvate dehydrogenase complex (PDC‐E2) and branched chain 2‐oxo acid dehydrogenase (BCOAD‐E2), independently of gender and age.

Spearman’s rho test				Multivariable logistic regression analysis^a^		
Autoantibodies	Variable^b^	*R*	*p*	Independent variables	OR (95% CI)	*p*
Anti‐p4.33 Ab	nPDC	0.403	<0.001	Anti‐p4.33 Ab	1.14 (1.03–1.20)	0.001
	PDC‐E2	0.408	<0.001	Gender	NA^c^	0.999
	BCOAD‐E2	0.391	<0.001	Age^d^	0.96 (0.91–1.02)	0.203
Anti‐Ap1‐17 Ab	nPDC	0.300	0.016	Anti‐Ap1‐17 Ab	1.00 (0.99–1.00)	0.428
	PDC‐E2	0.292	0.019	Gender	NA^c^	0.997
	BCOAD‐E2	0.423	<0.001	Age^d^	0.97 (0.93–1.01)	0.167
Anti‐Ap1‐17/17‐30 Ab	nPDC	0.152	0.229	Anti‐Ap1‐17/17‐30 Ab	1.00 (1.00–1.00)	0.094
	PDC‐E2	0.110	0.389	Gender	NA^c^	0.999
	BCOAD‐E2	0.251	0.045	Age^d^	0.96 (0.92–1.01)	0.162

Abbreviations: CI, confidence interval; NA, not applicable; nPDC, naïve pyruvate dehydrogenase complex; OR, odds ratio.

^a^Autoantibodies were analyzed by multivariable logistic regression, each variable being adjusted for gender and age, based on the outcome of AMA positivity.

^b^AMA levels were expressed as dot‐spot intensity score.

^c^AMA was not detected in any male patients.

^d^At diagnosis.

**Figure 3:**
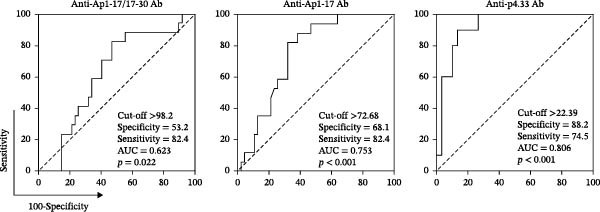
Receiver operating characteristic (ROC) analysis to define the best cut‐off level of anti‐CENP‐A Abs to Ap1‐17/17‐30, Ap1‐17, and p4.33 to discriminate AMA^pos^ (*n* = 17) from AMA^neg^ (*n* = 47) SSc patients. Cut‐off levels are expressed as the percentage of binding compared to the positive control. Area under the curve (AUC), sensitivity, specificity, and exact *p*‐values are indicated.

To establish the optimal cut‐off values for anti‐CENP‐A Ab subgroups able to discriminate AMA^pos^ from AMA^neg^ patients, ROC curves were generated (Figure 3). The results indicated a cut‐off of 98.2% for anti‐Ap1‐17/17‐30, 72.68% for anti‐Ap1‐17 Abs, and 22.39% for anti‐p4.33 Abs. As depicted in Figure 3, anti‐p4.33 Ab positivity showed a better performance in discriminating AMA status (AUC = 0.806, 88.2 specificity, 74.5 sensitivity, *p* < 0.001) as compared with anti‐Ap1‐17/17‐30 (AUC = 0.623, 53.2 specificity, 82.4 sensitivity, *p* = 0.015) and anti‐Ap1‐17 (AUC = 0.753, 68.1 specificity, 82.4 sensitivity, *p* < 0.001) Ab.

In line with these findings, the results of the multivariable analysis demonstrated that among anti‐CENP‐A subsets, anti‐p4.33 Ab positivity was the only variable associated with AMA positivity independently of gender and age at diagnosis (OR = 1.14; *p* = 0.001) (Table 2).

### 3.2. Identification of the Motif Sequence Shared by Peptide p4.33 With CENP‐A and Mitochondrial Antigens

Given the presence of AMA cross‐reacting with BCOADC‐E2 mitochondrial antigen in six anti‐p4.33^pos^ patients (lanes #5–9, 11; Figure 2A), we evaluated whether the antigenic motif shared by CENP‐A and PDC‐E2 was also expressed by the mitochondrial antigen BCOADC‐E2. As shown in Table 3, multiple sequence alignment of CENP‐A, PDC‐E2, BCOADC‐E2, and peptide p4.33 identified the amino acid motif “kPsaP.” A Swiss‐Prot database search showed that the motif was expressed by human viruses or bacteria. Specifically, the motif “kPsaP” was expressed by viral proteins encoded by herpes simplex virus 2 and human papillomavirus 35 and by proteins encoded by some bacteria, including *Chlamydia pneumoniae* and *Helicobacter pylori* (HP) (Table 4).

**Table 3:** Epitope shared between CENP‐A, and the E2 components of pyruvate dehydrogenase complex (nPDC) and branched chain 2‐oxo acid dehydrogenase (BCOAD‐E2).

Denomination	Amino acid sequence
CENP‐A^1–17^	MGPRRRSR **KP** E **AP** RRRS
PDC‐E2^331–347^	PTPQPLAPT **PSAP** CPAT
p4.33	‐‐‐‐GIAK **KPSAP** LQR‐
BCOADC‐E2^226–215^	‐‐‐‐MIEV **KPS** P **P** LIA
Motif	**-------**‐k**P**s**aP---**

*Note:* Bold amino acids indicate the residues corresponding to the shared epitope.

**Table 4:** Viral and bacterial pathogens containing the antigenic motif kPsaP recognized by anti‐p4.33 antibodies.

Pathogens^a^	Protein	Amino acid stretch containing motif (starting and ending position)
Viruses		
HHV‐2	Large tegument protein deneddylase	341–345
Human papillomavirus 35	Major capside protein L1	428–432
Bacteria		
*Chlamydia pneumoniae*	Chromosomal replication initiator protein DnaA2	87–91
*Clostridium botulinum*	Penicillin‐binding protein 1A	275–279
	Protein translocase subunit SecA	800–804
*Corynebacterium jeikeium*	Sec‐indipendent protein translocase protein TatB	183–187
*Coxiella burnetii*	RNA polymerase sigma factor RpoD	75–79
*Helicobacter pylori*	Aconitate hydratase B	362–366
*Mycobacterium leprae*	Thioesterase TesA	89–93
*Mycobacterium marinum*	UvrABC system protein C	291–295
*Nocarbioides* sp.	Large ribosomal subunit (50S) protein bL17	121–125
*Proteus mirabilis*	Putative minor fimbrial subunit PmfF	134–138
	Translation initiation factor IF‐2	184–188
	NAD kinase 2	298–302
*Vibrio cholerae* serotype O1	DNA topoisomerase 1	287–291
*Vibrio vulnificus*	Glucose‐1‐phosphate adenylyltransferase 1	177–181
*Yersinia pseudotuberculosis*	Voltage‐gated ClC‐type chloride channel ClcB	78–82
*Yersinia pestis*	Voltage‐gated ClC‐type chloride channel ClcB	78–82

Abbreviation: HHV‐2, Human herpesvirus 2.

^a^Sequences were retrieved from Swiss‐Prot Protein Sequence Database.

## 4. Discussion

The present exploratory retrospective study has shown that, by means of a p4.33 peptide‐based ELISA, it is possible to define a subgroup of anti‐CENP^pos^ patients with a significantly higher prevalence of AMA, who may warrant closer clinical monitoring for possible evolution toward PBC. Specifically, anti‐p4.33^pos^ patients displayed a markedly higher frequency of AMA compared with anti‐p4.33‐negative patients, supporting the potential role of these Abs in patient stratification within the anti‐CENP‐A‐positive SSc population.

The high prevalence of anti‐Ap1‐17 Abs observed in our cohort is not an unexpected finding and is consistent with previous data demonstrating a high percentage of anti‐CENP‐A Abs within the CENP^pos^ sera [17, 20].

Although AMA positivity alone is not sufficient for a definitive PBC diagnosis, some evidence suggests that the presence of AMA in patients with no symptoms and normal liver biochemistry may be strongly suggestive of PBC [21–24]. Indeed, in an earlier study by Mitchison et al. [21], liver biopsies from 24 of 29 AMA^pos^ patients with normal alkaline phosphatase (ALP) were consistent with PBC. During a median follow‐up of 17.8 years, 22 patients ultimately developed PBC symptoms, while 24 patients had abnormal liver function. In four of the nine patients who underwent biopsy at follow‐up, a progression of the histological grade was found [22]. Similar findings were obtained by Sun et al. [25], who demonstrated that liver biopsy revealed a histological diagnosis of PBC in 55 (82.1%) of 67 AMA^pos^ patients with normal ALP. A prospective study on 59 biochemically normal AMA‐positive individuals demonstrated in these subjects a progressive increase in immunological and liver tissue abnormalities moving towards a PBC spectrum [26]. Thus, the incidental identification of AMA in SSc patients with a normal biochemical profile offers the opportunity for a closer follow‐up for PBC, eventually, and its earlier diagnosis and for appropriate therapeutic intervention to improve patients’ outcomes. Indeed, the European Association for the Study of the Liver recommends follow‐up of patients with normal serum liver tests who are AMA^pos^ by annual biochemical reassessment for the presence of liver disease. Previous studies have demonstrated that, in SSc, anti‐CENP^pos^ are more often associated with PBC [10, 11, 27, 28]. In this study, PBC was diagnosed in 3 of the 15 anti‐p4.33^pos^ patients (20%). The presence of anti‐sp100 and ‐gp210 Abs in 3 out of the 12 anti‐p4.33^pos^ patients without a diagnosis of PBC suggests that all these patients may develop PBC. Indeed, previous studies have demonstrated that the transition from early to later stages of PBC is characterized by the presence of Abs reacting to a broader spectrum of mitochondrial and nuclear antigens, namely, sp100, gp120, and CENP A/B [26, 29]. No PBC diagnosis was made in the two AMA^pos^/anti‐p4.33^neg^ patients. It should be noted that, in this study, only clinically diagnosed PBC cases were recorded, and biochemical data, including ALP levels, as well as ANA IIF patterns beyond ACA reactivity, were not systematically available for all patients. This precluded any correlation analysis between anti‐p4.33 positivity and cytoplasmic reticular patterns, which were reported earlier by Ceribelli et al. [30] to identify AMA‐positive rheumatic patients, particularly those with SSc, even in the absence of liver dysfunction. We previously demonstrated that anti‐p4.33 Abs recognize an antigenic motif common to CENP‐A and PDC‐E2. Here, we have shown that the peptide p4.33 also shares sequence similarity with BCOADC‐E2, identifying a conserved “kPsaP” motif that may represent a common structural element recognized by anti‐p4.33 Abs. The motif PxxP, expressed by all the proteins investigated, is known to be expressed by viral proteins and is used to interact with cellular proteins containing the SH3 domain by molecular mimicry [31]. Also, molecular mimicry has been proposed as a pathogenic mechanism in the induction of anti‐CENP‐A [17, 32] and AMA [32–35] in SSc and PBC, respectively. Here, we found that the motif kPsaP was expressed by the viruses Herpes simplex virus 2 and Human papillomavirus 35 and by several bacteria, including *Chlamydia pneumoniae* and HP. Interestingly, the latter has been implicated in the pathogenesis of both SSc [36–38] and PBC [39, 40], based on the observations that a high prevalence of anti‐HP Abs has been reported in both diseases as compared to controls. Moreover, HP infection was associated with increased disease severity and activity in SSc [41, 42], and its eradication improved clinical symptoms and disease activity [43]. However, it should be emphasized that no clinical data regarding infection history or vaccination status were available in our cohort. Therefore, the proposed link between infectious and anti‐p4.33 autoreactivity remains speculative. Another limit to this study includes the low number of sera tested for AMA and the lack of data related to the follow‐up for some of the SSc/PBC/patients. Prospective studies will evaluate the predictive value of anti‐p4.33 Abs for PBC development and the possible role of pathogen infection, including HP, in triggering anti‐p4.33 Abs in SSc.

## 5. Conclusions

This study demonstrates that positivity to anti‐p4.33 Ab defines a subset of anti‐CENP‐Ab patients with a higher prevalence of AMA positivity. It also identifies a common motif shared by p4.33 and the mitochondrial antigens PDC‐E2 and BCOADC‐E2; this motif is expressed by certain pathogens and may trigger the production of autoantibodies [44].

## Author Contributions

Conceptualization, data curation, writing – original draft preparation, project administration: Elvira Favoino and Federico Perosa. Investigation, writing – review and editing: Elvira Favoino, Silvia De Santis, Sabina Piccolo, Giovanna Barbuti, Vasiliki Liakouli, Marta Vomero, Luca Navarini, Marcella Prete, Patrizia Leone, Vito Racanelli, Rosa Daniela Grembiale, Francesco Ciccia, Piero Ruscitti, Roberto Giacomelli, and Federico Perosa. Methodology: Elvira Favoino and Silvia De Santis. Funding acquisition: Federico Perosa.

## Funding

This work was supported by the Ministry for Universities and Research (MUR) PRIN 2022 PNRR grant (Grant P2022PKWF9) and European Union‐NextGeneration EU, Mission 4, Component 2, CUP (Grant H53D23007500001). Open access publishing facilitated by Universita degli Studi di Bari Aldo Moro, as part of the Wiley ‐ CRUI‐CARE agreement.

## Disclosure

All authors have read and agreed to the published version of the manuscript. The funder had no role in study design, data collection and analysis, decision to publish, or preparation of the manuscript. An abstract related to some of the results reported in this manuscript was accepted for poster presentation at the EULAR 2025 Congress and published in the conference abstract supplement. The abstract is cited in the References section.

## Ethics Statement

The study was conducted according to the guidelines of the Declaration of Helsinki and approved by the Ethical Committee of the University of Bari Medical School (No. 6017/C.E., 25/11/2019). Informed consent was obtained from all subjects included in the study.

## Conflicts of Interest

The authors declare no conflicts of interest.

## Data Availability

The data that support the findings of this study are available from the corresponding author upon reasonable request.
